# Add-on plasmonic patch as a universal fluorescence enhancer

**DOI:** 10.1038/s41377-018-0027-8

**Published:** 2018-07-04

**Authors:** Jingyi Luan, Jeremiah J. Morrissey, Zheyu Wang, Hamed Gholami Derami, Keng-Ku Liu, Sisi Cao, Qisheng Jiang, Congzhou Wang, Evan D. Kharasch, Rajesh R. Naik, Srikanth Singamaneni

**Affiliations:** 1 Department of Mechanical Engineering and Materials Science, Institute of Materials Science and Engineering, Washington University in St. Louis, St Louis, MO 63130 USA; 2 Department of Anesthesiology, Washington University in St. Louis, St. Louis, MO 63110 USA; 3 Siteman Cancer Center, Washington University in St. Louis, St. Louis, MO 63110 USA; 4 Department of Biochemistry and Molecular Biophysics, Washington University in St. Louis, St. Louis, MO 63110 USA; 5 The Center for Clinical Pharmacology, St. Louis College of Pharmacy and Washington University School of Medicine, St. Louis, MO USA; 6 711th Human Performance Wing, Air Force Research Laboratory, Wright-Patterson Air Force Base, Dayton, OH 45433 USA

## Abstract

Fluorescence-based techniques are the cornerstone of modern biomedical optics, with applications ranging from bioimaging at various scales (organelle to organism) to detection and quantification of a wide variety of biological species of interest. However, the weakness of the fluorescence signal remains a persistent challenge in meeting the ever-increasing demand to image, detect, and quantify biological species with low abundance. Here, we report a simple and universal method based on a flexible and conformal elastomeric film with adsorbed plasmonic nanostructures, which we term a “plasmonic patch,” that provides large (up to 100-fold) and uniform fluorescence enhancement on a variety of surfaces through simple transfer of the plasmonic patch to the surface. We demonstrate the applications of the plasmonic patch in improving the sensitivity and limit of detection (by more than 100 times) of fluorescence-based immunoassays implemented in microtiter plates and in microarray format. The novel fluorescence enhancement approach presented here represents a disease, biomarker, and application agnostic ubiquitously applicable fundamental and enabling technology to immediately improve the sensitivity of existing analytical methodologies in an easy-to-handle and cost-effective manner, without changing the original procedures of the existing techniques.

## Fluorescence: Stick-on patch fixes weak bio-signals

Adding a stretchy, nanoparticle-embedded elastomer onto standard fluorescence-based immunoassays raises biomarker signal intensities by two orders of magnitude. Nanomaterials such as gold nanorods have strong surface electromagnetic fields that can couple to the emission of a biomolecule’s fluorescent labels and enhance them, but only at certain distances apart. Srikanth Singamaneni, Rajesh Naik, and co-workers at the Washington University in St. Louis, U.S.A. have now put these nanoparticles into thin polydimethylsiloxane sheet to achieve optimal, atom-level contact for plasmonic enhancement on a variety of substrates. Because the patch is attached to immunoassays after analytical steps including antigen capture and fluorescent labeling have occurred, it avoids interfering with well-established technology. As an example, the team showed that biomarkers for kidney injury and disease could be detected at exceptionally low femtogram concentrations in urine samples.

## Introduction

Fluorescence probes and fluorometric approaches have been ubiquitously employed in biomedical research, not only as imaging tools for the visualization of the location and dynamics of cells and of various sub-cellular species and molecular interactions in cells and tissues but also as labels in fluoroimmunoassays for the detection and quantification of molecular biomarkers. Fluorescence-based techniques have radically transformed biology and life sciences by unraveling the genomic, transcriptomic, and proteomic signatures of disease development, progression, and response to therapy^1–3^. However, the occurrence of a “feeble signal” has been a persistent and recurring problem in the battery of detection and imaging techniques that rely on fluorescence. Overcoming this fundamental challenge without the use of specialized reagents, equipment, or significant modifications to well-established procedures is a holy grail in the field of biomedical optics. For example, there is an urgent need for ultra-sensitive fluoroimmunoassays that can be broadly adopted by most biological and clinical laboratories for the detection of target biological species with low abundance.

Improving the signal-to-noise ratio of the assays without deviating from the existing assay protocols will also relax the stringent requirements of high sensitivity and bulky photodetectors, shorten the overall assay time, lower the cost of implementation, eliminate cross-laboratory cross-platform inconsistency, and potentially propel these technologies to use in point-of-care, in-field, and resource-limited settings. Various techniques, including multiple-fluorophore labeling^4^, rolling cycle amplification^5,6^, and photonic crystal enhancement^7^, have been introduced to improve the signal-to-noise ratio of fluorescence-based imaging and sensing techniques. Despite the improved sensitivity, these technologies have not been widely adopted in research and clinical settings because most of them require significant modifications to the existing practices such as additional steps that significantly prolong the overall operation time, the need for specialized and expensive read-out systems, non-traditional data processing and analysis, or the use of temperature-sensitive reagents, which usually require tightly controlled transport and storage conditions.

Plasmonics has been recognized as a simple and highly effective approach for enhancing fluorescence. Enhancement of the emission of fluorophores in close proximity to plasmonic nanostructures is attributed to the enhanced electromagnetic field (local excitation field) at the surface of the plasmonic nanostructures and the decrease in the fluorescence lifetime due to the coupling between the excited fluorophores and the surface plasmons of the nanostructures^8–17^. To date, various plasmonic substrates, such as periodic gold arrays^18,19^ and metal nanoislands^11–14^, have been shown to give rise to strong fluorescence enhancement. Although these plasmonic surfaces are highly attractive, their real-world application, for example, in fluoroimmunoassays, has been limited. The limited application of plasmon-enhanced fluoroimmunoassays in research and clinical settings is due to several factors: (i) Most of the existing techniques require the fluoroimmunoassay to be performed on pre-fabricated substrates, typically a rigid glass slide with metal nanostructures deposited on it, instead of standard or sometimes irreplaceable bioanalytical platforms (e.g., 96-well plates and nitrocellulose membranes), which significantly limits the broad applicability of the techniques; more importantly, the requirement for the use of special substrates limits cross-platform and cross-laboratory consistency and seamless integration with widely employed bioanalytical procedures, representing a major bottleneck for the exploitation of conventional plasmon-enhanced fluorescence. (ii) Non-traditional bioconjugation procedures, complex interactions between biomolecules and metal nanostructures, and poor stability of biomolecules (e.g., antibodies) immobilized on metal surfaces not only complicate the assay procedures but also impose further technical challenges in their widespread application^20^. Thus, it is imperative to address these challenges to propel the plasmon-enhanced fluorescence techniques to practical applications.

Here, we introduce a simple, universal, and “add-on” fluorescence enhancement technique based on a “plasmonic patch” that can be applied on various fluorescent surfaces to achieve large and uniform fluorescence enhancement. To the best of our knowledge, this work represents the first demonstration of flexible plasmonics for fluorescence enhancement. In stark contrast with the existing plasmon enhancement techniques, which require significant modifications of the existing fluoroimmunoassay methods, the plasmonic patch approach demonstrated here requires virtually no change of the existing protocols except for the addition of the “patch” as the new, final step. Due to the enhanced electromagnetic field, the plasmonic patch can efficiently enhance the fluorescence by up to 100 times, leading to an ~300-fold increase in assay sensitivity. More importantly, the plasmonic patch exhibits excellent stability and low cost and entails the use of an extremely user-friendly protocol. This represents a “ready-to-use” technique that can be integrated with current biomedical research and clinical infrastructure to generate immediate results and impact.

## Materials and methods

### Fabrication of a plasmonic patch

Sylgard 186 (Dow Corning) polydimethylsiloxane (PDMS) elastomer was mixed at a 10:1 (base to curing agent) ratio. The prepolymer was spin-coated at 3000 rpm for 30 s on a polystyrene dish with a diameter of 3.5 cm. PDMS was then cured at 70 °C for 15 h. Once cured, PDMS was treated with oxygen plasma for 3 mins and subsequently immersed into 0.2% aqueous poly(styrene sulfonate) (PSS) solution for 20 mins. PSS treatment gave rise to a negative charge on the surface of the PDMS film, facilitating the absorption of positively charged plasmonic nanoparticles through electrostatic interactions. Plasmonic nanoparticle solution was centrifuged and redispersed into a specific volume of nanopure water (for details, please see the Supporting Information). PSS-treated PDMS was incubated with the plasmonic nanoparticle solution for 15 h in dark conditions. Subsequently, PDMS was rinsed with nanopure water and blow dried with nitrogen, leaving a surface with uniformly adsorbed plasmonic nanoparticles.

### Polymer spacer on a plasmonic patch

Eight microliters of (3-aminopropyl)trimethoxysilane (APTMS) and the desired amount of trimethoxypropylsilane (TMPS (0–8 µl)) (for details, please see the Supporting Information) were added to 3 ml of phosphate-buffered saline (1× PBS). The plasmonic patch was incubated in the above solution for 2 h. After 2 h, the plasmonic patch was rinsed with PBS and nanopure water followed by blow drying with nitrogen gas.

### Fluorescence-linked immunosorbent assay with a plasmonic patch

Fluorescence-linked immunosorbent assay was first implemented using 96-well plates with a glass bottom (Cellvis). The glass surface of each well was treated to achieve aldehyde functionality. The subsequent procedures were identical to those of enzyme-linked immunosorbent assay (ELISA) (R&D Systems (DY1750B, DY1757)) until the streptavidin binding step. Instead of HRP-labeled streptavidin, 100 µl of dye-labeled streptavidin (CW800 or LT680 (LICOR)) was diluted to the final concentration of 50 ng/ml using a reagent diluent and added to each well, followed by a 20-min incubation. A plasmonic patch was subsequently transferred to each well of the 96-well plate. The LICOR Odyssey CLx scanner was used to scan the 96-well plate. For the fluorescence-linked immunosorbent assay performed using plastic bottom 96-well plates, the procedure remained the same except for the omission of the surface modification steps.

### Fluorescence enhancement on a protein microarray

Commercialized protein microarray chip kits were purchased from RayBiotech (Custom G-Series Antibody Array, AAX-CUST-G). Antibodies were printed on a glass slide with four subarrays available per slide. The slide was blocked by a blocking buffer (in kit) for 30 mins. Patients’ and volunteers’ urine samples were diluted twice using the blocking buffer, and 90 µl of the diluted samples was added into each sub-well of the microarray chip, followed by a 2-h incubation at room temperature. The chip was then washed thoroughly with the wash buffer (in kit). Seventy microliters of biotin-conjugated anti-cytokines (in kit) was added to each subarray, and the chip was incubated at room temperature with gentle shaking. After 2 h, the chip was washed, 70 µl of streptavidin-CW800 (100 ng/ml in blocking buffer, LICOR) was added, and the plate was incubated under dark conditions for 20 mins. The chip was washed thoroughly first with wash buffer and then with nanopure water and was then blow dried under nitrogen gas. The glass chip was scanned using a LICOR Odyssey CLx scanner. A plasmonic patch was cut into 1 × 1 cm^2^ pieces and applied to the top of each subarray, followed by the attachment of a gold-coated reflective film with the same dimensions.

## Results and discussion

**Fig. 1:**
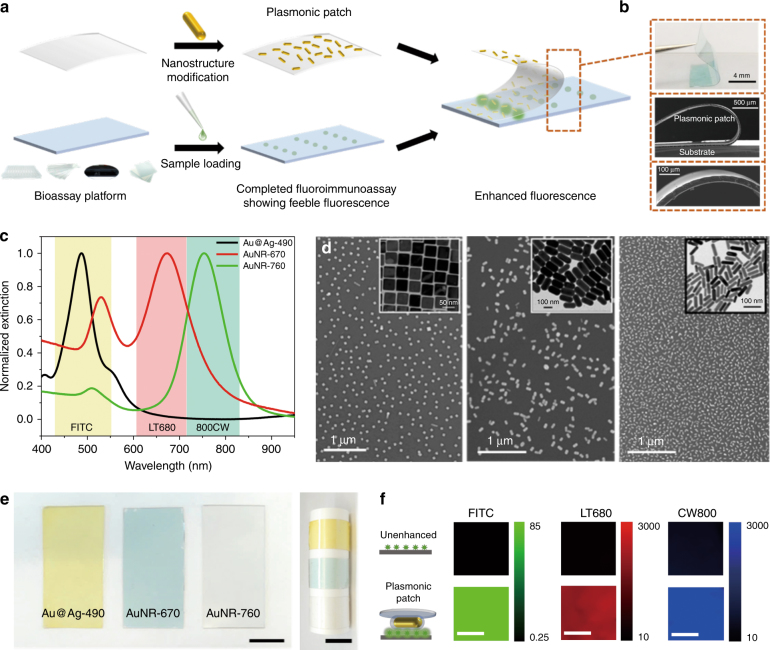
Plasmonic patch fabrication and material characterization. **a** Schematic illustration of the fabrication of a plasmonic patch and its application in fluoroimmunoassays. A large enhancement in the fluorescence signal is simply achieved by the transfer of the plasmonic patch onto a surface with fluorescent species. This “add-on” step does not change the well-established procedures of current fluoroimmunoassays and can thus be seamlessly integrated with a variety of existing assays to significantly enhance their fluorescence. **b** Top: Photograph showing the transfer of a plasmonic patch to a planar surface. Middle: SEM image demonstrating the flexibility, as well as conformability to the substrate, of the plasmonic patch. Bottom: SEM image of the cross-section of the plasmonic patch showing an average thickness of 30 µm. **c** Normalized extinction spectra of aqueous solutions of the three representative plasmonic nanostructures employed in this study (from left to right: Au@Ag-490, AuNR-670, and AuNR-760). The extinction bands of Au@Ag-490, AuNR-670, and AuNR-760 exhibit significant overlap with the absorption bands (excitation source) of FITC, LT680, and 800CW, respectively. **d** SEM images of the plasmonic patch surface revealing the uniform distribution of plasmonic nanostructures on PDMS (from left to right: Au@Ag-490, AuNR-670, and AuNR-760). Insets show representative TEM images of the corresponding plasmonic nanostructures. **e** Photograph of plasmonic patches modified with various nanostructures (left). The flexibility of the plasmonic patch is further demonstrated by rolling it around a cylindrical support (right). The scale bar represents 1 cm. **f** Fluorescence map of three fluorophores adsorbed on a silicon substrate in the presence and absence of a plasmonic patch (left scale bar represents 10 µm; middle and right scale bars represent 1 mm)

We introduce a novel material platform, namely, a “plasmonic patch,” for the enhancement of fluorescence on arbitrary surfaces. The fluorescence enhancement demonstrated here involves the transfer of a plasmonic patch, a transparent elastomeric film with adsorbed rationally designed metal nanostructures, onto a fluorescent surface to achieve conformal contact (Fig. 1a). The plasmonic nanostructures on the elastomeric film come into close proximity to the fluorescent species on the surface, resulting in a large and uniform enhancement of the fluorescence.

### Plasmonic patch fabrication and material characterization

A thin PDMS layer (~30 µm thick) is employed as the “patch” material due to its high mechanical flexibility (elastic modulus ~1 MPa) (Fig. 1b), optical transparency (>95% transmittance within the wavelength range of 400–900 nm)^21^, excellent processability, and low cost^22^. The elastomeric nature of the PDMS enables conformal contact (down to the atomic level) of the patch with diverse surfaces, which is critical for fluorescence enhancement because the enhanced electromagnetic field of the plasmonic nanostructures is limited to the first few nanometers from the metal surface^23^. The plasmonic patch can be tailored for a specific fluorophore by maximizing the overlap between the localized surface plasmon resonance (LSPR) of the nanostructures and the optical absorption (excitation source) of the fluorophore to achieve the highest enhancement^24,25^. As representative examples, we fabricated plasmonic patches using three distinct nanostructures: (i) gold core-silver shell nanocubes (Au@Ag nanocubes) with an LSPR wavelength of 490 nm (Au@Ag-490 henceforth, edge length ~48.5 nm) and gold nanorods (AuNRs) with a longitudinal LSPR wavelength of (ii) 670 nm (AuNR-670 henceforth, length ~112.2 nm, diameter ~54.5 nm) and (iii) 760 nm (AuNR-760 henceforth, length ~62.7 nm, diameter ~18.1 nm) (Fig. 1c). Scanning electron microscopy (SEM) images indicate a highly uniform distribution of the plasmonic nanostructures on the PDMS film, with no sign of aggregation or patchiness (Fig. 1d), ensuring nanoscale conformal contact between the plasmonic patch and the surface of interest. Extinction spectra obtained from the plasmonic patches further validate the absence of aggregates (Figure S1). The final density of the plasmonic nanostructures on the PDMS was determined to be 31/µm^2^ for Au@Ag-490, 21.4/µm^2^ for AuNR-670, and 169/µm^2^ for AuNR-760. The flexible plasmonic patches exhibit a distinct and uniform color corresponding to the LSPR wavelength of the nanostructures (Fig. 1e). The three plasmonic patches described above were designed for fluorescein isothiocyanate (FITC) (Au@Ag-490), LT680 (AuNR-670), and 800CW (AuNR-760), chosen in this study as representative fluorophores. Transfer of the corresponding plasmonic patches to silicon surfaces coated with FITC, LT680, and 800CW resulted in a uniform enhancement of the fluorescence from these surfaces (Fig. 1f). Additionally, the transfer process is easy, and its implementation does not require special training for users (Figure S2). The fluorescence intensity in the presence of a plasmonic patch was found to be nearly 50 times higher than that obtained from an unenhanced surface under identical illumination conditions (Figure S3). In addition to silicon, we applied plasmonic patches to glass, nitrocellulose, and polystyrene (a common material for microtiter plates) surfaces, which are extensively employed in various fluorescence-based detection, quantitative sensing, and imaging techniques. The excellent conformality of the plasmonic patch with all of the above materials resulted in large fluorescence enhancements of the dyes deposited on these surfaces. The intensity cross-section profiles obtained for these different materials demonstrate up to 80-fold enhancement in the fluorescence from the regions with the plasmonic patch (center) compared to unenhanced regions (periphery) (Figure S4).

### Distance-dependent fluorescence enhancement and spacer layer

**Fig. 2:**
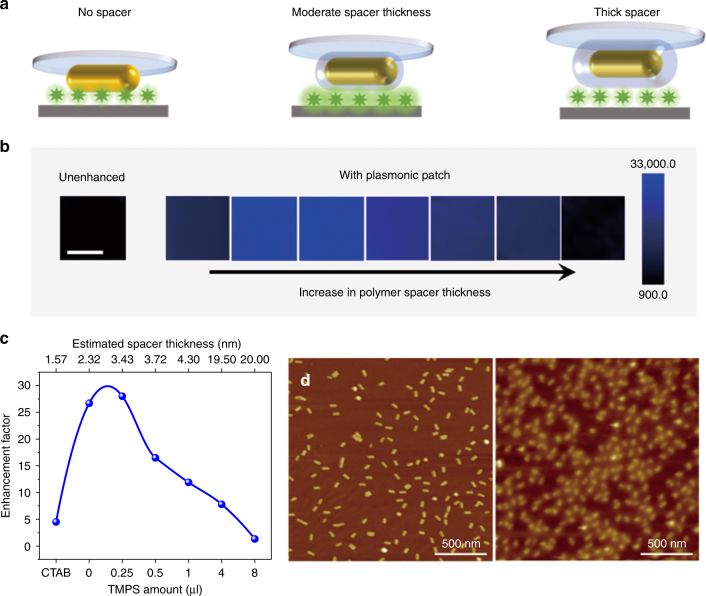
Distance-dependent fluorescence enhancement and spacer layer. **a** Schematic illustration showing a plasmonic patch with a polymer layer acting as a spacer between fluorophores and plasmonic nanostructures. The spacer thickness is optimized to achieve the maximum enhancement efficiency. **b** Fluorescence map of CW800 in the presence of plasmonic patches with increasing spacer layer thickness (TMPS and APTMS volume ratio is 0:0, 0:8, 0.25:8, 0.5:8, 1:8, 4:8, and 8:8 from left to right). **c** Fluorescence enhancement factor as a function of the spacer thickness (TMPS amount in the polymerization process). **d** AFM images of pristine Au nanorods (left) and Au nanorods with a polymer spacer (right) (TMPS and APTMS volume ratio of 4:8)

It is known that the evanescent nature of the enhanced electromagnetic field at the surface of plasmonic nanostructures results in a highly distance-dependent enhancement of Raman scattering and fluorescence at the surface of the plasmonic nanostructures^26–30^. When fluorophores are brought in direct contact (or in extremely close proximity) to plasmonic nanostructures, non-radiative energy transfer between the fluorophore and metal surface results in fluorescence quenching^31^. On the other hand, an increase in the distance between the fluorophores and metal nanostructures results in a decrease in the enhancement due to the decay in the electromagnetic field from the surface of the nanostructures. Taken together, these effects mean that an optimal distance between the metal surface and fluorophore is critical to ensure the maximum enhancement^32^. To achieve an optimal distance between the plasmonic nanostructures and fluorophores of interest, we employed a polysiloxane copolymer film formed on the surface of the plasmonic patch as a spacer layer (Fig. 2a). TMPS and APTMS, which are hydrolytically unstable, were copolymerized onto the plasmonic patch composed of AuNR-760. The two monomers underwent rapid hydrolysis and subsequent condensation, yielding an amorphous copolymer layer (Figure S5A)^33^. An increase in the thickness of the spacer layer resulted in a gradual redshift of the longitudinal LSPR wavelength of AuNRs due to the increase in the refractive index of the medium surrounding the nanostructures (Figure S5B). We estimated the thickness of the spacer layer for the different TMPS amounts used during the polymerization (see Supporting Information for an estimation of the spacer thickness, Figure S5C–I). With the increase in the spacer layer thickness, we observed a steep increase in the fluorescence enhancement efficacy of CW800 followed by a relatively shallow reduction (Fig. 2b, c). Atomic force microscopy (AFM) images depicted the morphology change of the plasmonic patch after the formation of the polysiloxane layer, which further confirmed the uniform deposition of the polymer spacer onto the AuNRs (Fig. 2d). Plasmonic patches with the optimal spacer layer were used in the subsequent studies (described below).

### Patterned plasmonic patch and localized fluorescence enhancement

**Fig. 3:**
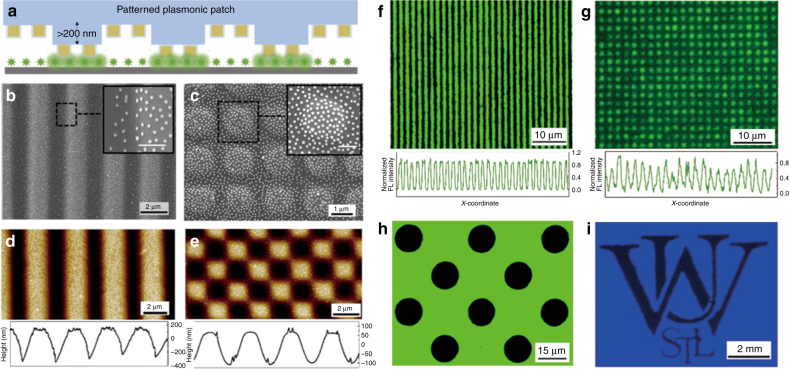
Patterned plasmonic patch and localized fluorescence enhancement. **a** Schematic showing a patterned plasmonic patch, which selectively enhances the fluorescence in the regions of conformal contact. In all of the used patterns, the height of the surface-relief portions is >200 nm. SEM image of a (**b**) stripe array and (**c**) square lattice PDMS with Au@Ag-490 adsorbed (insets show zoomed-in SEM images of the highlighted area revealing a uniform distribution of the plasmonic nanostructures on both elevated and surface-relief regions; inset scale bars represent 500 µm). AFM images of (**d**) stripe array (z scale: 430 nm) and (**e**) square lattice (z scale: 200 nm) plasmonic patches revealing the height profile of the surfaces. Fluorescence images of an FITC-coated silicon surface with (**f**) stripe array and (**g**) square lattice plasmonic patches on top. The plots below reveal the fluorescence intensity profiles. **h** Fluorescence map of FITC with a plasmonic patch (with Au@Ag-490 adsorbed) with circular pores. **i** Fluorescence image (CW800) of the “Washington University in St. Louis” logo obtained using a plasmonic patch (with AuNR-760 adsorbed) with an engraved logo

To demonstrate that the fluorescence enhancement induced by the “plasmonic patch” is localized to areas that are in conformal contact with the plasmonic patch, we employed a patterned patch layer with well-defined surface-relief structures on both microscales and macroscales. Transfer of the patterned plasmonic patch onto a silicon substrate with uniformly adsorbed fluorophores resulted in conformal contact between the raised regions of the plasmonic patch and the substrate, while the surface-relief regions remained far from the substrate (Fig. 3a). As representative microscale structures, we employed plasmonic patches with a stripe array and a square lattice composed of Au@Ag-490 (Fig. 3b, c). Insets of the SEM images depict the uniform adsorption of the nanostructures in both the elevated and surface-relief regions of the microstructured PDMS surface. AFM images reveal that the depth of the ridges in the stripe array are ~400 nm (Fig. 3d). The square lattice array, on the other hand, is composed of three regions with distinct heights (pores, struts, and nodes with increasing height) (Fig. 3e). After the transfer of the patterned plasmonic patch onto silicon coated with FITC, the plasmonic patch exhibited selective enhancement of fluorescence from the raised regions of the plasmonic patch that came into conformal contact with the silicon surface. In the case of the plasmonic patch with the stripe pattern, the fluorescence image shows arrays of bright and dark stripes corresponding to the raised and surface-relief regions of the plasmonic patch, respectively (Fig. 3f). Notably, the fluorescence enhancement in the case of the square array is confined to nodes, indicating that the struts and pores are too far from the surface to enhance the fluorescence (Fig. 3g). In addition to micropatterns, we also fabricated a plasmonic patch with a feature size ranging from tens of microns to millimeters (Fig. 3h, i and Figure S6). Transfer of plasmonic patches engraved with a square array of circular holes (with Au@Ag-490) and the “Washington University in St. Louis” logo (with AuNR-760) resulted in fluorescence images with a square array of dark circles and the logo with high image quality and feature fidelity (Fig. 3h, i).

### Plasmonic patch-enhanced fluoroimmunoassays

**Fig. 4:**
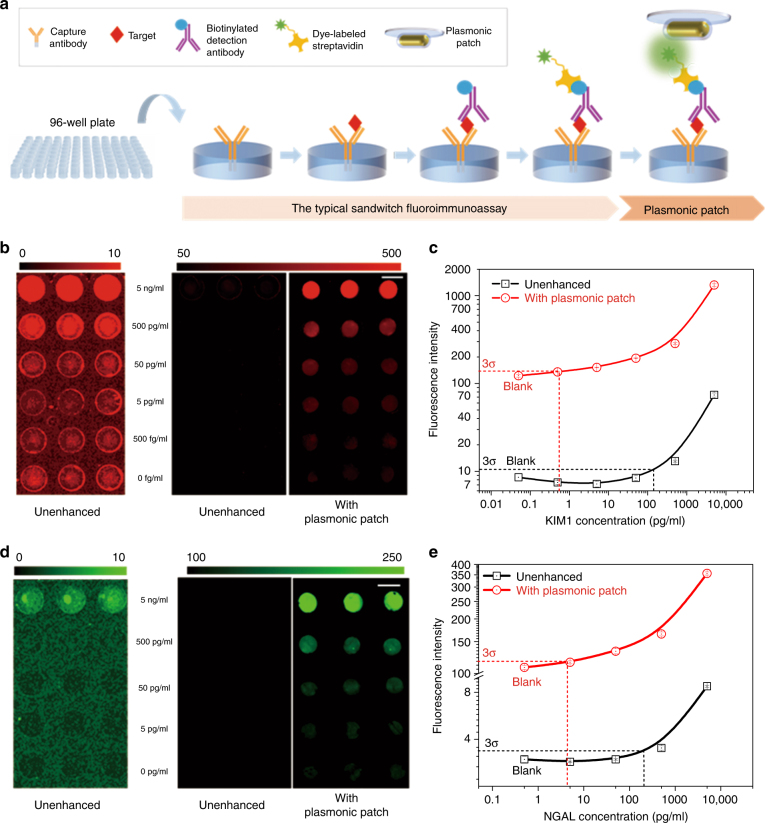
Plasmonic patch-enhanced fluoroimmunoassays. **a** Schematic showing the concept of a plasmonic patch-enhanced fluoroimmunoassay implemented in a glass bottom 96-well plate, demonstrating the wide applicability of the plasmonic patch. Fluorescence intensity maps of fluoroimmunoassays corresponding to different concentrations of (**b**) KIM1 and (**d**) NGAL, two early-stage biomarkers for acute kidney injury (AKI) and chronic kidney disease (CKD) (left and middle images show the unenhanced assays corresponding to the different color scales shown in the figures; right image shows the plasmonic patch-enhanced assay revealing a large enhancement in the fluorescence signal as well as a broadened dynamic range compared to the unenhanced assay (scale bar represents 5 mm)). Plot showing the fluorescence intensities corresponding to different concentrations of (**c**) KIM1 and (**e**) NGAL. The limits of detection identified in the plots show ~300-fold and ~100-fold improvement for KIM1 and NGAL, respectively, compared to the unenhanced assay

We now turn our attention to the application of the plasmonic patch as a universal fluorescence enhancer in fluoroimmunoassays. A typical sandwich fluoroimmunoassay involves the following major steps: (i) capture of the target antigen by an immobilized antibody; (ii) binding of the biotinylated detection antibody to the captured antigen; and (iii) binding of fluorescently labeled streptavidin (Fig. 4a). We hypothesize that the addition of a plasmonic patch after the last step (i.e., binding of the fluorescently labeled streptavidin) can result in a large enhancement of the fluorescence intensity and significantly improve the limit-of-detection (LOD given by the average fluorescence intensity at zero concentration (blank) plus three times its standard deviation). To verify this hypothesis, we implemented a fluoroimmunoassay in a heterogeneous, solid-phase format by using a 96-well microtiter plate as a sampling platform, a standard assay format extensively employed in bioanalytical research and clinical diagnostics (Fig. 4a).

We used two early-stage biomarkers for acute kidney injury (AKI) and chronic kidney disease (CKD), namely, kidney injury molecule-1 (KIM1) and neutrophil gelatinase-associated lipocalin (NGAL), as representative examples for probing the efficacy of the plasmonic patch in improving the bioanalytical parameters of fluoroimmunoassays^34–36^. The assays were first implemented on a 96-well plate with a glass bottom. In the KIM1 immunoassay, we used LT680 as the fluorescence tag and the plasmonic patch based on AuNR-670 as the enhancer. To probe the enhancement in the sensitivity and LOD, serial dilutions of KIM1 of known concentrations (5 ng/ml to 500 fg/ml) in PBS with 1% bovine serum albumin (BSA) were employed as standards. Fluorescence images obtained after the application of the plasmonic patch revealed a strong enhancement in the fluorescent intensity compared to that obtained prior to the application of the plasmonic patch (Fig. 4b). The fluorescence signal from the unenhanced (pristine) spots was detectable only for the two highest concentrations (5 and 0.5 ng/ml) (Fig. 4b, left and middle images). On the other hand, the fluorescence signal with the plasmonic patch could be detected down to 500 fg/ml (Fig. 4b). The concentration–response plot revealed a 36-fold enhancement in the fluorescence intensity with the plasmonic patch compared to the unenhanced signal (Fig. 4c). The LOD (3*σ*) values of the unenhanced and plasmon-enhanced KIM1 assays were determined to be 140 and 0.5 pg/ml, respectively, representing a 280-fold improvement in the LOD. Consequently, the enhanced KIM1 assay exhibited a three orders of magnitude higher dynamic range compared to the unenhanced assay. The fluorescence signal after the application of the plasmonic patch exhibited excellent stability even after 4 weeks of storage under dark conditions (Figure S7). To demonstrate the broad applicability of the plasmon-enhanced fluoroimmunoassay, we used NGAL as another representative example. CW800 (conjugated to streptavidin) was used as the fluorescence label to demonstrate the tunability of the plasmonic patch. Following the transfer of the plasmonic patch, we observed a fluorescence enhancement of up to 103 times and an ~100-fold lower LOD compared to the unenhanced NGAL assay (Fig. 4d, e). Consistently, the NGAL assay implemented on a common 96-well plate with a plastic bottom (instead of a glass bottom) also exhibited a strong fluorescence enhancement in the presence of the plasmonic patch (Figure S8), further validating the plasmonic patch as a substrate material-agnostic technology.

ELISA is widely employed in clinical and research settings and is often considered as the “gold standard” for protein biomarker detection and quantification. We compared the performance of the plasmon-enhanced fluoroimmunoassay with ELISA using KIM1 as a representative biomarker. In addition to simplifying the overall assay procedure (e.g., obviating the need for enzymatic amplification), the LOD of the plasmon-enhanced fluoroimmunoassay was found to be ~30 times lower (0.5 pg/ml) than that of ELISA (15.6 pg/ml) (Fig. 4c and Figure S9). Notably, the dynamic range of the enhanced fluoroimmunoassay spanned five log orders of KIM1 concentration, while the dynamic range of ELISA was only 2.5 log orders of KIM1 concentration (Fig. 4c and Figure S9). The higher dynamic range of the enhanced fluoroimmunoassay is expected to enable the quantification of a wider range of biomarker concentrations in human urine samples, as described below.

**Fig. 5:**
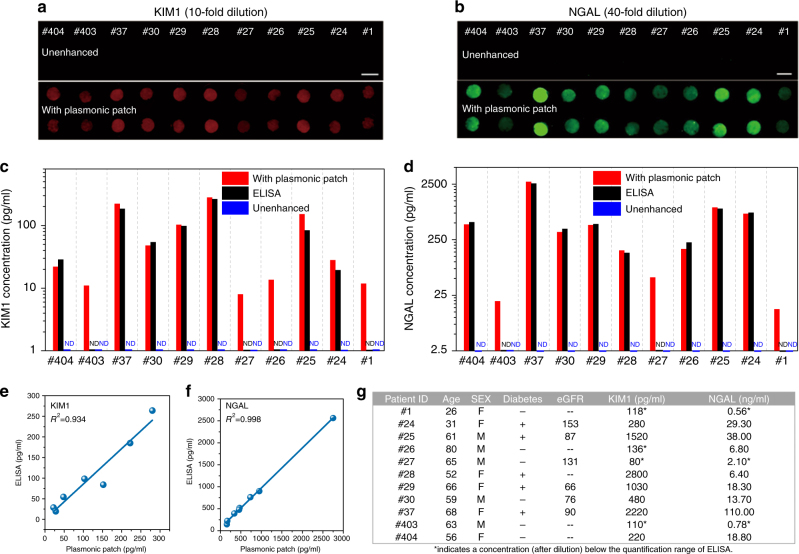
Analysis of urine samples from patients and self-described healthy volunteers. Fluorescence intensity maps of (**a**) KIM1 (10-fold dilution) and (**b**) NGAL (40-fold dilution) immunoassay for urine samples from eight patients (ID: #24, #25, #26, #27, #28, #29, #30, and #37) and three self-described healthy volunteers (ID: #1, #403, and #404). (Top: Unenhanced fluorescence map. Bottom: Plasmonic patch-enhanced fluorescence map. Scale bar = 5 mm.) **c** KIM1 and (**d**) NGAL concentrations in the urine samples (diluted 10-fold for KIM1 and 40-fold for NGAL) as determined by unenhanced fluorescence assay, plasmon-enhanced fluorescence assay, and ELISA. Plot showing the correlation between the concentration of (**e**) KIM1 and (**f**) NGAL determined using ELISA and plasmonic patch-enhanced fluorescence assay. **g** Table summarizing the age, sex, diabetic condition, and estimated glomerular filtration rate (eGFR), and measured (by the plasmon patch-enhanced method) urinary concentrations of KIM1 and NGAL in urine samples from 11 patients or healthy volunteers

Following the successful demonstration of the plasmonic patch-enhanced fluoroimmunoassay, we set out to analyze urine samples from patients and self-described healthy volunteers in order to determine the concentrations of KIM1 and NGAL. To demonstrate the wide applicability of the technique, we implemented KIM1 and NGAL fluoroimmunoassays on glass and plastic bottom 96-well plates, respectively. The urine samples were diluted with 1% BSA in PBS to minimize the confounding from inter-individual differences in urine pH and solute content. For KIM1 (10-fold dilution) and NGAL (40-fold dilution), the plasmon-enhanced fluoroimmunoassay exhibited a dramatic increase in the fluorescence compared to the unenhanced fluoroimmunoassay (Fig. 5a (KIM1) and Fig. 5b (NGAL)). The enhanced fluorescence signal was used to quantify the biomarker concentration in the urine samples. We also used standard ELISA to determine the KIM1 and NGAL concentrations in the human urine samples. The concentrations of the biomarker in urine determined by the above three assays (unenhanced and enhanced fluoroimmunoassays and ELISA) are compared in Figure 5c (KIM1) and Fig. 5d (NGAL). The unenhanced fluoroimmunoassay was not able to detect KIM1 or NGAL in any of the human urine samples. In stark contrast, the plasmon-enhanced fluoroimmunoassay was able to quantify both KIM1 and NGAL concentrations in all human urine samples, some of which were even lower than the LOD of ELISA. For the samples that were quantifiable using both ELISA and enhanced fluoroimmunoassay, the concentration of the biomarker determined using the enhanced fluoroimmunoassay showed excellent agreement with that determined using “gold standard” ELISA for both KIM1 (*r*^2^ = 0.934) and NGAL (*r*^2^ = 0.998) (Fig. 5e, f).

The biomarker concentrations in the human urine samples were determined by accounting for the dilutions in each of the assays, and the results are presented in Figure 5g. The estimated glomerular filtration rate (eGFR) determined from the serum creatinine concentration is the standard metric of kidney function^37^. eGFR decreases to below 90 (ml/min) as the kidney function declines^37^. The two urine biomarkers can provide diagnostic kidney disease information beyond that of eGFR. NGAL and KIM1 concentrations in healthy humans are <20 and <1 ng/ml, respectively. In AKI, NGAL exceeds 100 ng/ml^36,38,39^. Taking patients #24 and #37 as examples, while their eGFR levels (153 and 90 ml/min) are within the normal range, their NGAL and KIM1 concentrations were significantly higher, indicating a high risk of chronic kidney disease (#24) and AKI (#37). Notably, for diabetics, the eGFR levels tend to increase to 150 ml/min followed by a significant decrease (down to 30 ml/min) with time. The higher eGFRs of patients #24 and #37 and their slightly elevated KIM1 and NGAL concentrations may be due to the patients being diabetic, which is a risk factor for chronic kidney disease (Fig. 5g)^40^.

### Application of a plasmonic patch on a protein microarray

**Fig. 6:**
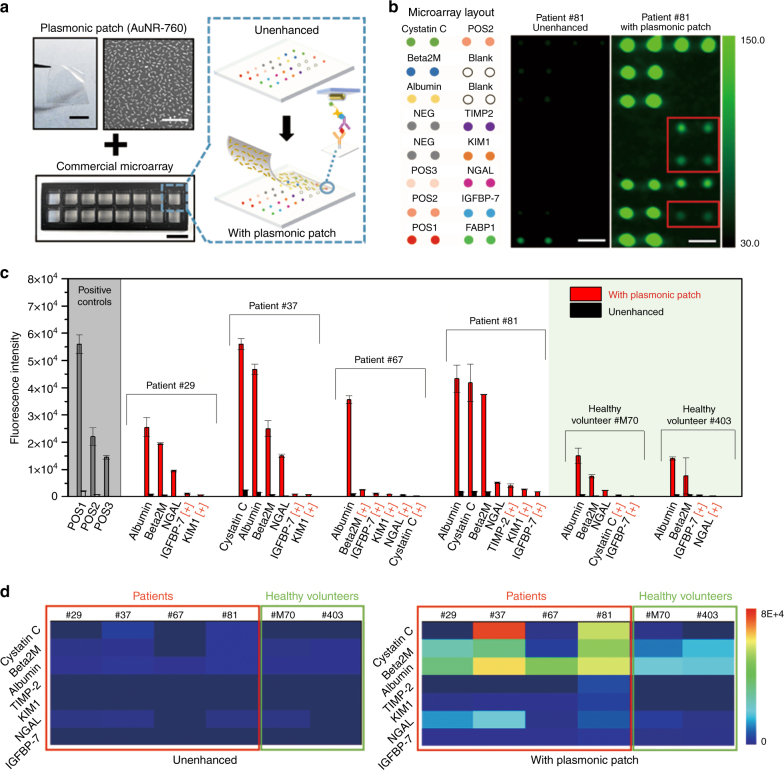
Application of a plasmonic patch on a protein microarray. **a** Illustration showing the application of a plasmonic patch to enhance the bioanalytical parameters of a protein microarray. Left: Photograph depicting the plasmonic patch employed for enhancing the fluorescence of a protein microarray (scale bar represents 5 mm), SEM image demonstrating the uniform absorption of AuNR-760 on the PDMS surface (scale bar represents 500 nm), and photograph of a commercial protein microarray substrate with 16 sub-wells for simultaneous analysis of multiple samples (scale bar represents 1 cm). Right: Schematic showing the concept of a plasmonic patch-enhanced microarray, which enables a highly sensitive profiling of eight AKI and CKD biomarkers simultaneously. **b** Left: Schematic showing the layout of antibodies printed on the bottom of each sub-well. Middle: Unenhanced fluorescence intensity map representing the AKI and CKD protein biomarker profile of patient #81. Right: Fluorescence map generated after the application of the plasmonic patch (scale bar represents 400 µm). **c** Quantitative measurements of fluorescence intensity corresponding to the concentrations of various biomarkers in the urine samples of four patients (ID: #81, #67, #37, and #29) and two self-described healthy volunteers (ID: #M70 and #403). [+] indicates biomarker detected only after the application of the plasmonic patch. POS spots in the microarray represent three distinct positive control signal intensities (POS1 > POS2 > POS3). **d** Fluorescence intensity heat map corresponding to the concentrations of kidney disease biomarkers in the urine samples of four patients (ID: #81, #67, #37, and #29) and two healthy volunteers (ID: #M70 and #403) before (left) and after (right) the application of the plasmonic patch

To demonstrate the applicability of the plasmonic patch in enhancing the sensitivity of immuno-microarrays, we customized a microarray of antibodies to the biomarkers of kidney injury as a representative example to test the performance of the plasmonic patch in a multiplexed and high-throughput biosensing platform (Fig. 6a). This microarray is composed of eight capture antibodies corresponding to the AKI and CKD protein biomarkers, printed in duplicate on a glass slide isolated by a plastic frame with a feature diameter of 150 µm. Biotinylated immunoglobulin Gs of three gradient concentrations were printed in duplicate as positive controls (Fig. 6b, left schematic showing the protein layout on the microarray). Six human urine samples were diluted twofold using a blocking buffer and added to each sub-well on the glass slide. Subsequently, the captured biomarker proteins were exposed to a biotinylated detection antibody cocktail followed by exposure to CW800-labeled streptavidin. The conventional microarray procedure ends at this step, at which point the biomarker concentration is quantified by analyzing the localized fluorescent signal on the respective antibody spot. The enhanced assay demonstrated here involves the addition of a 1 × 1 cm^2^ plasmonic patch modified with AuNR-760 on top of each array (see Methods for details).

The fluorescence map from a single sample (patient #81, Fig. 6b, right panels) is informative. In addition to the large enhancement of the weak fluorescence of albumin, cystatin-C, β_2_ microglobulin (Beta 2M), and NGAL in the unenhanced microarray, the plasmonic patch enabled the detection and quantification of analytes that could not be detected at all by the conventional method (red boxes in Figure 6b). These new analytes are tissue inhibitor of metalloproteinases 2, KIM1, and insulin-like growth factor-binding protein 7, which are specific and important biomarkers for early detection of AKI^27,41^. In addition to patient #81, the plasmonic patch consistently enhanced the fluorescence signals of the microarray exposed to urine samples from patients #29, #37, and #67 and healthy volunteers #M70 and #403 (Figure S10). Quantitative measurement of the antibody spot intensity from the urine of the six individuals showed 20-fold to 137-fold increase in the fluorescence of several analytes and the detection of other analytes enabled only by the enhancement from the plasmonic patch (Fig. 6c, the [+] mark indicates that the biomarker is only detected with the plasmonic patch). Comparison between the unenhanced and plasmonic patch-enhanced fluorescence heat maps from the six donors further revealed the high signal-to-noise ratio and a broadened dynamic range (Fig. 6d).

## Conclusions

Most previous plasmon-enhanced fluorescence assays rely on engineering the substrate to be plasmonically active through either the deposition of metal islands or adsorption of plasmonic nanostructures. These methods naturally require the utilization of special surfaces and possibly significant alterations of the read-out devices and the bioassay protocol. Here, we demonstrated an alternative method in which the enhancement is achieved by a simple transfer of a plasmonic patch onto a surface with fluorescent species. This novel approach not only obviates the need for special substrates or tedious bioconjugation procedures but also offers excellent tunability of the plasmonic properties (over the entire visible and near-infrared wavelength range) and distance between the metal surface and fluorophores. Notably, the magnitude of the fluorescence enhancement obtained using plasmonic substrates described in the past is highly dependent on the size of the capture antibody, antigen, and detection antibody that exist between the plasmonic nanostructures on the substrate and the fluorophores. The enhancement is therefore dictated by the preset “biological spacer,” leaving little control over the key design parameter for maximum enhancement, namely, the spacer layer thickness. By contrast, as an “add-on-top” layer, the plasmonic patch demonstrated here enables complete control over the distance between the plasmonic nanoantennas and fluorescent species. The facile control of the spacer thickness ensures the highest fluorescence enhancement despite the variations in the immunofluorescent assays, which is especially important in multiplexed platforms.

We also demonstrated the application of this platform technology in enhancing the bioanalytical parameters (sensitivity, LOD, and dynamic range) of fluoroimmunoassays implemented in a standard 96-microplate format and an antibody microarray. The plasmonic patch consistently resulted in a more than two orders of magnitude fluorescence intensity enhancement, leading to an ~300-fold lower LOD and a three orders of magnitude higher dynamic range. The improvement in the bioanalytical parameters was found to be consistent across different assay formats, target biomarkers, and fluorophores. Significantly, this method can be implemented with existing bioassays without any modification of the standard operating procedures, additional operational training, or modification of the read-out devices. As a part of the rigorous validation of this technology, we analyzed urine samples from patients and healthy volunteers. Unlike the unenhanced fluoroimmunoassay and ELISA, the plasmon-enhanced fluoroimmunoassay enabled the detection and quantification of low concentration biomarkers from all patients and healthy volunteers. The added sensitivity of the plasmon-enhanced assay enables facile quantification of the biomarkers with low abundance and provides physiological and pathological information that is often missed by the conventional immunoassays.

Multiplexed microarrays based on fluorescence are extensively employed in expression profiling, drug-target binding assays, and high-throughput proteomics^42,43^. Compared to a singlex platform, such as ELISA, the technique presented here allows researchers and clinicians to examine a large number of biomarkers in parallel to achieve patient stratification and monitoring of multifactorial diseases with a limited sample volume, thereby minimizing the assay cost and time for the performance of multiple individual biomarker assays. Moreover, high-throughput profiling of the biomarkers enables personalized medicine with holistic, molecular fingerprinting of diseases, accommodating greater diagnostic resolution between closely related disease phenotypes^44^. The sensitivity and specificity for the diagnosis of kidney disease have been proven to be significantly greater when combining the urinary levels of multiple biomarkers compared to the use of individual biomarkers^36^. However, despite the availability of various commercialized products, this multiplexed methodology suffers from inferior sensitivity and relatively high LOD compared to ELISA, which hinders its widespread application.

The plasmonic patch demonstrated here overcomes the above-mentioned challenges and provides a path forward for broad application of multiplexed microarrays. We have demonstrated the application of the plasmonic patch in the multiplexed detection of a panel of biomarkers for kidney diseases. Our results suggest that the plasmonic patch could significantly enhance the ability to elucidate low-level kidney function parameters (biomarkers) to provide holistic kidney disease information. Notably, the better performance of the multiplexed microarray originates from the extremely simple “patch transfer” process, which does not alter the established process flow of immuno-microarrays. Additionally, this technique represents an inexpensive approach for the enhancement of fluorescence, and the cost for one piece of plasmonic film (1 × 1 cm^2^) was estimated to be approximately US$0.05. We expect that this easily deployed technique could be seamlessly applied to a broad range of multiplexed platforms in diagnostics, proteomics, and genetics to address the unmet need for a greater signal intensity.

Our work here has primarily focused on the introduction of the plasmonic patch concept and on demonstrating its application in the enhancement of the bioanalytical parameters of fluoroimmunoassays implemented in microtiter plates and microarrays. However, it is important to note that this technique has broad implications in bioimaging, blotting, histology, and virtually any other application involving fluorescence. Due to the minimal perturbation of the standard materials and procedures, this novel technique can be readily adapted to a number of different fluorescence-based technologies to alleviate the waste of resources arising from facility update, reduce the assay cost and time, eliminate cross-platform inconsistency, and potentially propel these technologies to use in point-of-care, in-field and resource-limited settings.

## Electronic supplementary material

supplemental informaiton
