# Supplementary material for: Add-on plasmonic patch as a universal fluorescence enhancer
Source: Light Sci Appl. 2018 Jul 4;7:29. doi: 10.1038/s41377-018-0027-8 (PMC6107004; doi:10.1038/s41377-018-0027-8)
Supplement: Supplementary file 1 — supplemental informaiton [file 41377_2018_27_MOESM1_ESM.docx]

**Supporting Information:** **Add-on Plasmonic Patch as a Universal Fluorescence Enhancer**

Jingyi Luan^1^, Jeremiah J. Morrissey^2,3^, Zheyu Wang^1^, [Hamed Gholami Derami](http://pubs.rsc.org/en/results?searchtext=Author%3AHamed%20Gholami%20Derami)^1^, Keng-Ku Liu^1^, Sisi Cao^1^, Qisheng Jiang^1^, Congzhou Wang^1^, Evan D. Kharasch^2,3,4,5^, Rajesh R. Naik^6*^, Srikanth Singamaneni^1,3*^

^1^Department of Mechanical Engineering and Materials Science, Institute of Materials Science and Engineering, Washington University in St. Louis, St Louis, MO, 63130, USA.

^2^Department of Anesthesiology, Washington University in St. Louis, St. Louis, MO, 63110, USA

^3^Siteman Cancer Center, Washington University in St. Louis, St. Louis, MO, 63110

^4^Department of Biochemistry and Molecular Biophysics, Washington University in St. Louis, St. Louis, MO, 63110, USA. ^5^The Center for Clinical Pharmacology, St. Louis College of Pharmacy and Washington University School of Medicine, St. Louis, MO, USA

^6^711th Human Performance Wing, Air Force Research Laboratory, Wright-Patterson Air Force Base, Dayton, OH 45433

* To whom correspondence should be addressed: [singamaneni@wustl.edu](mailto:singamaneni@wustl.edu)；rajesh.naik@us.af.mil

***Methods:***

***Synthesis of Au@Ag-490:*** To synthesize Au@Ag-490 nanocubes, Au nanospheres with a diameter of 30 nm were employed as the seed. The 30 nm Au nanospheres were in turn synthesized by a seed-mediated method.^1^ First, Au seeds were synthesized by adding 0.6 ml of ice-cold NaBH_4_ solution (10 mM) into a solution containing 0.25 ml HAuCl_4_ (10 mM) and 9.75 ml CTAB (0.1 M) under vigorous stirring at room temperature for 10 min. The solution color changed from yellow to brown indicating the formation of Au seed. Next, 0.25 ml of the seed solution was added to a growth solution containing 10 ml of CTAC (0.2 M) and 7.5 ml of ascorbic acid (0.1 M) under stirring. Ten milliliters of HAuCl_4_ (0.5 mM) was added to the growth solution as a single addition, resulting in the formation of Au nanospheres with a diameter of 10 nm. The 10 nm Au nanospheres were centrifuged at 13,000 rpm for 30 minutes. For further growth of Au nanoparticles to a diameter of 30 nm, a growth solution comprised of 30 ml CTAC (0.1 M) and 1.95 ml ascorbic acid (10 mM) was prepared. To the resulting solution, 1.2 ml of 10 nm Au nanospheres (extinction 1.0) was added under stirring. A total 30 ml solution of HAuCl_4_ (0.5 mM) was added into the above mixture at the rate of 0.5 ml min^-1^ under stirring. After the reaction was completed, the solution containing 30 nm Au nanospheres was centrifuged at 8000 rpm for 10 min and redispersed into nanopure water to achieve a final extinction ~1.2.

As-synthesized 30 nm Au nanospheres (1.5 ml) and 13.5 ml of CTAC (20 mM) were mixed under stirring at 60 °C for 20 min. Subsequently, 1.5 ml of AgNO_3_ (2 mM), 3.75 ml of CTAC (20 mM), and 0.7 ml of ascorbic acid (100 mM) were added under stirring at 60 °C, and the solution was left under stirring for 4 hours. After 4 hours, the as-synthesized Au@Ag-490 nanocubes were centrifuged (8000 rpm) and redispersed into 7.5 ml aqueous solution of CTAC (20 mM) and stored under dark conditions until use. The average edge length of the Au@Ag-490 nanocubes was measured to be 48±1.4 nm from TEM images.

***Synthesis of AuNR*:** AuNR was prepared by seed-mediated method.^2,3^ Au seed was synthesized by the method described above. For the synthesis of AuNR-760, the growth solution was prepared by the sequential addition of aqueous HAuCl_4_ (0.01 M, 2 ml), CTAB (0.1 M, 38 ml), AgNO_3_ (0.01 M, 0.4 ml), and ascorbic acid (0.1 M, 0.22ml) followed by gentle inversion to homogenize the solution. Subsequently, 48 µl of the seed solution was added into the growth solution and left undisturbed in dark for 12 hours. For AuNR-670, the growth solution contained HAuCl_4_ (0.01 M, 2 ml), CTAB (0.1 M, 40 ml), AgNO_3_ (0.01 M, 0.4 ml), HCl (1.0 M, 0.8 ml), and ascorbic acid (0.1 M, 0.32 ml). After the seed solution was diluted 50 times with nanopure water, 10 µl of the diluted seed solution was injected into the above growth solution and left undisturbed under dark for 12 hours. The obtained AuNR solutions were then subjected to anisotropic oxidation by adding H_2_O_2_ (30 wt%).^4,5^ The oxidation process was monitored by measuring the extinction spectra of the AuNR solution. When the longitudinal LSPR wavelength reached the desired value, the AuNRs were washed by two cycles of centrifugation with 0.1 M CTAB and finally redispersed in nanopore water. The final extinction of AuNR-670 and AuNR-760 was adjusted to be ~1.5.

***Fabrication of plasmonic patch:*** Sylgard 186 (Dow Corning) polydimethylsiloxane (PDMS) elastomer was mixed at a 10:1 (base to curing agent) ratio. 0.1 g of the prepolymer was spin coated at 3000 rpm for 30 seconds on a polystyrene petridish with a diameter of 3.5 cm. PDMS was then cured at 70 °C for 15 hours. Once cured, PDMS was treated with oxygen plasma for 3 mins and was subsequently immersed into 0.2% aqueous poly(styrene sulfonate) (PSS) solution for 20 mins. PSS treatment rendered a negative charge on the surface of PDMS film, which facilitates the absorption of positively charged plasmonic nanoparticles through electrostatic interaction. For adsorbing the plasmonic nanostructures onto the modified PDMS surface, nanoparticle solution (1 ml) was centrifuged and redispersed into a specific volume of nanopure water (1.5 ml nanopure water for AuNR and 4 ml for Au@Ag-490). The PSS treated PDMS was incubated with the plasmonic nanoparticles for 15 hours in dark conditions. Subsequently, PDMS was rinsed with nanopure water and blow dried with nitrogen, leaving a surface with uniformly adsorbed plasmonic nanoparticles.

***Polymer spacer on plasmonic patch:*** Eight microliters of APTMS and desired amount of TMPS (0 to 8 µl) were added into 3 ml of phosphate buffered saline (1X PBS). Plasmonic patch was incubated in the above solution for 2 hours. After 2 hours, the plasmonic patch was rinsed with PBS and nanopure water followed by blow drying with nitrogen gas. In terms of plasmonic patch of AuNR-760, 0.25 µl TMPS and 8 µl of APTMS were added into 3 ml of PBS for 2 hours incubation in order to obtain optimal enhancement. Plasmonic patch of AuNR-670 was modified with APTMS (8 µl APTMS in 3 ml PBS for 2 hours incubation) to form optimal polymer spacer. We adopted these optimal spacer conditions in the following experiments.

***Fabrication of patterned plasmonic patch:*** For microscale patterns, a silicon “master” was fabricated using standard photolithography procedure. Patterned PDMS film was fabricated by casting the PDMS on the silicon master with complimentary structures.^6^ The PDMS was cured at 70 °C for 15 hours and was then peeled off from the master. The surface of the patterned PDMS was modified with corresponding plasmonic nanoparticles through the method described above.

***Fluorescence enhancement using plasmonic patch:***

Aldehyde activation of silicon and glass substrates: Glass and silicon substrates were first cleaned using Piranha solution (3:1 concentrated sulfuric acid to 30% hydrogen peroxide solution) followed by thorough rinsing with nanopure water and blow drying with nitrogen gas (Caution: Piranha solution is extremely dangerous and proper care needs to be executed in handling and disposal). A mixture of 0.25 ml of (3-Aminopropyl)triethoxysilane (APTES) and 0.25 ml of nanopure water was added into 4.5 ml pure ethanol followed by gentle mixing. Cleaned silicon and glass substrates were incubated in the above APTES solution for 2 hours. The substrates were subsequently rinsed with nanopure water and then ethanol followed by blow drying under nitrogen gas. APTES-modified substrates were incubated in 2.5% glutaraldehyde (GA) solution (in PBS, pH=7.4) for 2 hours. The substrates were then rinsed with PBS followed by nanopure water to remove excess GA. The aldehyde activated substrates react with primary amines of the protein to form Schiff’s base linkage.

Plasmonic patch enhanced fluorescence: 96-well plate (polystyrene), nitrocellulose membrane (with an average pore size of 0.22 µm), aldehyde activated glass and silicon substrates were incubated in CW800 labeled anti-mouse IgG (10 ng ml^-1^ in PBS) under dark conditions for 8 hours. The substrates were then rinsed with PBS and nanopure water. Plasmonic patch with AuNR-760 was cut into a circular shape with a diameter of 4 mm using a skin biopsy punch. For silicon substrate, the plasmonic patch was directly applied on top of the wet surface and the sample was scanned after 20 mins to allow for the evaporation of the water between the plasmonic patch and the substrate. For the other three types of substrates, a reflective flexible film (polyacrylic film coated with 50 nm of gold layer using thermo-evaporator) was added on the top of plasmonic patch. Bottom reading mode was adopted for these three substrates due to their optical transparency (or translucence). All the fluorescent signals were obtained by LICOR Odyssey CLx imager.

***ELISA:*** Duoset ELISA from R&D systems (DY1750B, DY1757) were employed for the quantification of NGAL and KIM1, respectively. 96-well plastic plates were first coated with capture antibodies (For NGAL: 2 µg ml^-1^ in PBS; for KIM1: 4 µg ml^-1^ in PBS) through overnight incubation at room temperature, followed by blocking with 300 µl reagent diluent (PBS containing 1% BSA, 0.2 µm filtered). After three times washing with PSBT (0.05% Tween 20 in PBS), 100 µl of serial diluted standard samples as well as patients’ urine samples (10-fold dilution for KIM1, 40-fold dilution for NGAL) were added into different wells (all dilutions using reagent diluent). The plate was then covered with an adhesive strip and incubated at room temperature for 2 hours. After washing three times with PBST, the plate was incubated with biotinylated detection antibodies (For NGAL: 25 ng ml^-1^ in reagent diluent; for KIM1: 50 ng ml^-1^ in reagent diluent) at room temperature for 2 hours, washed another three times with PBST, incubated with HRP labeled streptavidin (200-fold dilution using reagent diluent) for 20 mins, and washed three times with PBST. 100 µl of substrate solution (1:1 mixture of Color Reagent A (H_2_O_2_) and Color Reagent B (Tetramethylbenzidine) (R&D Systems, Catalog # DY999)) was added to each well and the reaction was stopped by adding 50 µl of H_2_SO_4_ (2 N) (R&D Systems, Catalog # DY994) after 20 mins. Optical density of each well was determined immediately using a microplate reader set to 450 nm.

***Fluorescence-linked immunosorbent assay with plasmonic patch:*** Fluorescence-linked immunosorbent assay was first implemented using 96-well plates with glass bottom (Cellvis). The glass surface of each well was treated to achieve aldehyde functionality using the method described above. Subsequent procedures were identical with ELISA until the streptavidin binding step. Instead of HRP-labeled streptavidin, 100 µl of dye-labeled streptavidin (CW800 or LT680 (LICOR)) was diluted to a final concentration of 50 ng ml^-1^ using reagent diluent and added to each well followed by a 20-min incubation. Plasmonic patch was subsequently transferred to each well of the 96-well plate using the method introduced above. The LICOR Odyssey CLx scanner was used to scan the 96-well plate using 800 nm channel (intensity of 3.0, resolution of 84 µm, scanning height of 2 mm) and 700 nm channel (intensity of 3.5, resolution of 84 µm, scanning height of 2 mm). For the fluorescence-linked immunosorbent assay performed using plastic bottom 96-well plates, the procedure remained the same except the omission of surface modification steps. While scanning, the height was set to 4 mm for the plastic 96-well plate. All the fluorescent signals were analyzed by calculating the average intensity of the center (2 mm diameter) within each well.

***Fluorescence enhancement on protein microarray:*** Commercialized protein microarray chip kits were purchased from RayBiotech (Custom G-Series Antibody Array, AAX-CUST-G). Antibodies were printed on a glass slide with 4 subarrays available per slide. The slide was blocked by blocking buffer (in kit) for 30 mins. Patients’ and volunteers’ urine samples were diluted twice using blocking buffer and 90 µl of the diluted samples were added into each sub-well of the microarray chip followed by a two-hour incubation at room temperature. The chip was then washed thoroughly with wash buffer (in kit). Seventy microliters of biotin-conjugated anti-cytokines (in kit) were added to each subarray and the chip was incubated at room temperature with gentle shaking. After two hours, the chip was washed and 70 µl of streptavidin-CW800 (100 ng ml^-1^ in blocking buffer, LICOR) was added and the plate was incubated under dark conditions for 20 mins. The chip was washed thoroughly with wash buffer then nanopure water and blow dried under nitrogen gas. The glass chip was scanned by LICOR Odyssey CLx scanner using 800 nm channel (intensity of 2, resolution of 21 µm, scanning height of 1 mm). Plasmonic patch of AuNR-760 was cut into 1×1 cm^2^ and applied on the top of each subarray followed by attachment of gold coated reflective film of the same size. The chip was rescanned using the same settings. Median background signal was adopted for analyzing the spot intensity.

***Estimation of the spacer layer thickness:***

To estimate the thickness of the spacer layer, we employed the refractive index sensitivity of the localized surface plasmon resonance (LSPR) of AuNRs. The adsorbate (polymer) thickness-dependent LSPR wavelength shift of AuNR was determined using layer-by-layer (LbL) assembly of polyelectrolyte multilayers (PEM) on the surface of AuNR-760. Electrostatic LbL assembly was performed by alternate adsorption of positively charged poly(allyl amine hydrochloride) (PAH) and negatively charged poly(styrene sulfonate) (PSS). This method has been shown to provide linear growth of the polymer layer thickness with number of bilayers and excellent control over the thickness of polymer layers down to 1 nm.^7^ AuNR-760 was first treated with oxygen plasma to remove the surfactant (CTAB) layer on the top. As expected, the spectra of AuNR-760 revealed a progressive red-shift in LSPR wavelength with the deposition of each polyelectrolyte bilayer (PAH/PSS) due to the increase in the refractive index (from air to polymer layer, Figure S5C). AFM scratch test after the deposition of 10 bilayers revealed the thickness of the PEM on the glass surface between AuNR to be ∼20 nm (Figure S5D, E), which closely agrees with the value reported previously for the PAH/PSS system.^8^ Therefore, the thickness of one polyelectrolyte bilayer can be estimated to be ~2 nm. The cumulative LSPR shift could be fit using the following equation^9^:

$$R=m\Delta\eta(1-exp(-2d/l))$$

where $R$ is LSPR shift, $m$ is the refractive index sensitivity of AuNR, $\Delta\eta$ is the change in the refractive index in RIU, $d$ is the adsorbate layer thickness (thickness of the polyelectrolyte layer in this case), and $l$ is the EM decay length (Figure S5F).

Using this equation, we are able to correlate the LSPR shift induced by the addition of polymer spacer (compared to AuNR after removal of CTAB) to the spacer layer thickness (Figure S5G, H).

**Figure S1.**  Extinction spectra of plasmonic patches adsorbed with distinct plasmonic nanostructures (measured in air).


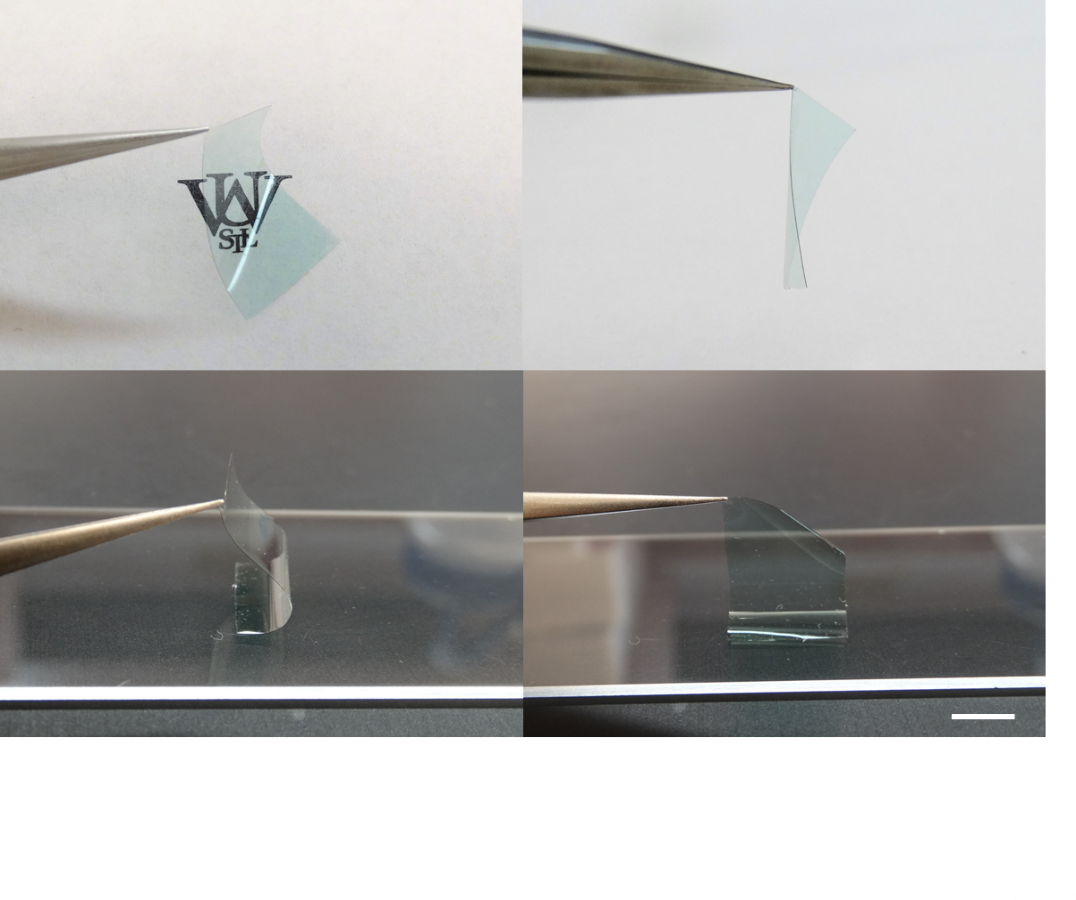


**Figure S2.**  Photos depicting the transfer process of and ease of handling of plasmonic patch (AuNR-670) using a sharp forceps. Scale bar represents 5 mm.

**Figure S3.**  Fluorescence spectra of (A) FITC, (B) LT680, and (C) CW800 adsorbed on silicon substrate in the presence and absence of plasmonic patch.

A

B

C

**Figure S4.** Plasmonic patch enhanced fluorescence of CW800 uniformly adsorbed on silicon, glass, nitrocellulose, and polystyrene surfaces. Circular plasmonic patch with AuNR-760 (with a diameter of 4 mm) is applied on each surface coated with CW800. Fluorescent intensity profile across the plasmonic patch reveals strong enhancement within the plasmonic patch region compared to the area outside the patch (75 times for silicon, 24 times for glass, 9 times for nitrocellulose, and 80 times for polystyrene). Inset fluorescence image in each plot shows the fluorescent map of the surface with plasmonic patch at the center (scale bar represents 1 mm).

A

B

C


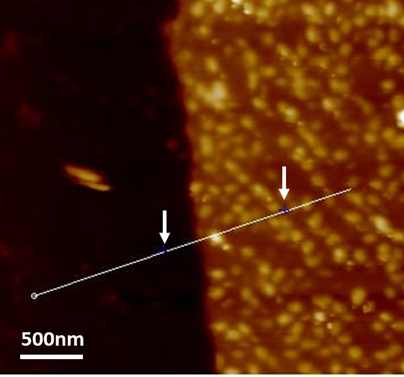


E

F

D


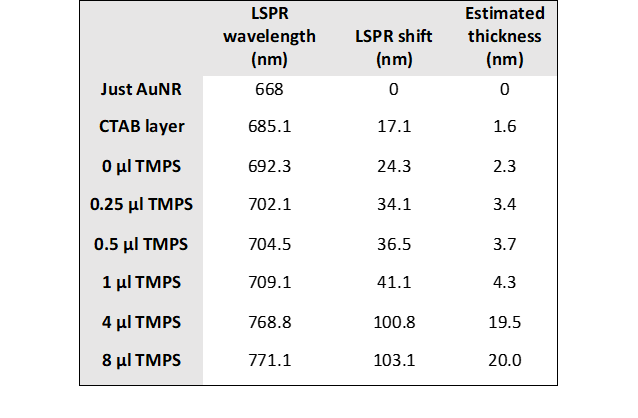


G

H

I

**Figure S5.**  (A) Schematic illustration showing the copolymerization of TMPS and APTMS, which is employed as a spacer layer on plasmonic patch. (B) LSPR wavelength of AuNR (with CTAB layer) and AuNR coated with siloxane copolymer. TMPS/APTMS (µl/µl) employed in the polymerization process from left to right: 0:0 (CTAB), 0:8, 0.25:8, 0.5:8, 1:8, 4:8, and 8:8. (C) UV−vis extinction spectra of AuNR-760 (after CTAB removal) following the deposition of each polyelectrolyte layer showing a progressive red-shift and increase in the intensity of the longitudinal plasmon band. (D) AFM image along the edge of an intentional scratch in polyelectrolyte multilayers (PEM) film comprised of 10 bilayers deposited on AuNR. (E) Cross-sectional profile shows the thickness of the PEM film on the glass substrate. (F) Plot of cumulative shift of longitudinal plasmon resonance wavelength with the deposition of PEM. (G) Fitted equation representing the relation between LSPR shift and absorbate thickness: *R = 117.7 (1 - exp(-0.1d))*. Using this equation, thickness of the spacer layer can be determined from its corresponding LSPR shift (marked by *). (H) Table summarizing the value of AuNR LSPR wavelength and corresponding LSPR shift under different spacer layer condition, with the calculated thickness of the spacer layer listed behind. (I) Fluorescence enhancement factor as a function of estimated spacer thickness.


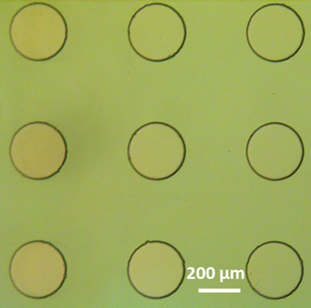


A

B

C


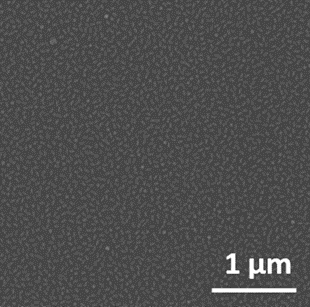

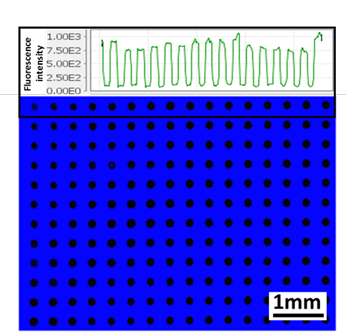


**Figure S6.** (A) Optical image showing PDMS patch with a square array of circular holes. (B) SEM image showing the uniform distribution of AuNR-760 on the patterned PDMS surface. (C) Fluorescence map of CW800 after transfer of patterned plasmonic patch (plot at the top shows the intensity profile of one row in the array).

**Figure S7.** Fluorescence intensity of plasmonic patch enhanced fluoroimmunoassay after being stored with the plasmonic patch for 2 and 4 weeks. Unlike colorimetric ELISA that shows rapid degradation in the optical intensity, plasmonic patch-enhanced fluoroimmunoassay exhibits a stable signal with no compromise in the LOD for up to 4 weeks.

A

B

With plasmonic patch

**Figure S8.** (A) Fluorescence intensity maps of NGAL fluoroimmunoassay implemented on a common 96-well plate with a polystyrene bottom. Left and middle images show the unenhanced assays which correspond to different color scales shown in the images. Right image shows the plasmonic patch enhanced assay revealing a large enhancement in the fluorescence signal (scale bar represents 5 mm). (B) Plot showing the calibration curve for plasmonic patch enhanced fluoroimmunoassay implemented on 96-well plate with a polystyrene bottom.

**Figure S9.** KIM-1 concentration -response curve obtained by ELISA. The LOD is determined to be 15.6 pg ml^-1^.


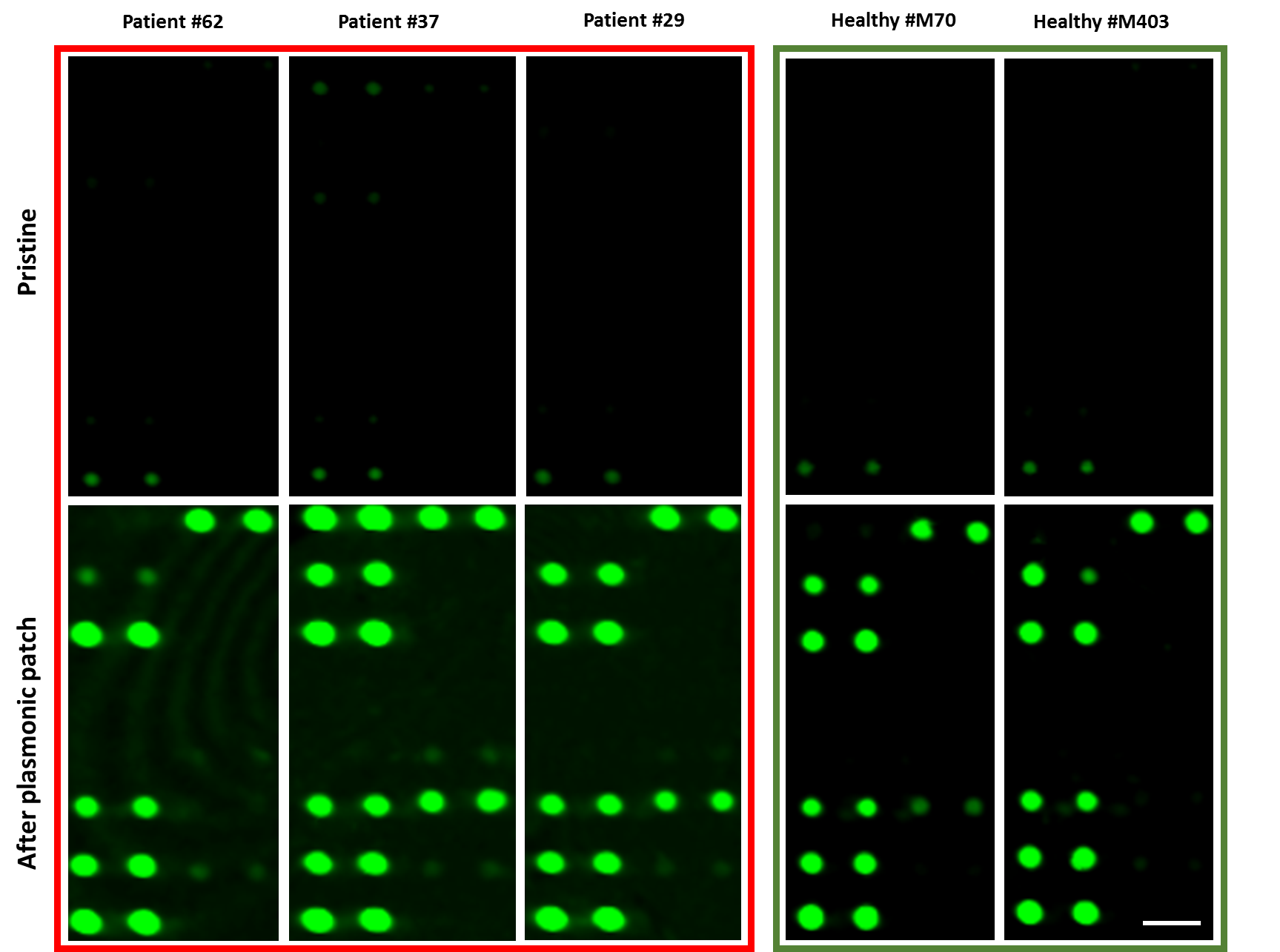


**Figure S10.** Unenhanced (top) and plasmonic patch enhanced (bottom) fluorescence intensity maps representing the kidney disease biomarker profile of patients (ID: # 67, #37, #29) and healthy volunteers (ID: #M70, #403) (scale bar represents 400 µm).

**References**

1 Zheng, Y., Zhong, X., Li, Z. & Xia, Y. Successive, Seed‐Mediated Growth for the Synthesis of Single‐Crystal Gold Nanospheres with Uniform Diameters Controlled in the Range of 5–150 nm. *Particle & Particle Systems Characterization* **31**, 266-273 (2014).

2 Lee, K.-S. & El-Sayed, M. A. Dependence of the enhanced optical scattering efficiency relative to that of absorption for gold metal nanorods on aspect ratio, size, end-cap shape, and medium refractive index. *The Journal of Physical Chemistry B* **109**, 20331-20338 (2005).

3 Gole, A. & Murphy, C. J. Azide-derivatized gold nanorods: functional materials for “click” chemistry. *Langmuir* **24**, 266-272 (2008).

4 Yuan, H. *et al.* Reshaping anisotropic gold nanoparticles through oxidative etching: the role of the surfactant and nanoparticle surface curvature. *RSC Advances* **5**, 6829-6833 (2015).

5 Carattino, A., Khatua, S. & Orrit, M. In situ tuning of gold nanorod plasmon through oxidative cyanide etching. *Physical Chemistry Chemical Physics* **18**, 15619-15624 (2016).

6 Qin, D., Xia, Y. & Whitesides, G. M. Soft lithography for micro-and nanoscale patterning. *Nature protocols* **5**, 491-502 (2010).

7 Kedem, O., Tesler, A. B., Vaskevich, A. & Rubinstein, I. Sensitivity and optimization of localized surface plasmon resonance transducers. *ACS nano* **5**, 748-760 (2011).

8 Tian, L., Chen, E., Gandra, N., Abbas, A. & Singamaneni, S. Gold nanorods as plasmonic nanotransducers: distance-dependent refractive index sensitivity. *Langmuir* **28**, 17435-17442 (2012).

9 Lu, G. *et al.* Anisotropic plasmonic sensing of individual or coupled gold nanorods. *The Journal of Physical Chemistry C* **115**, 22877-22885 (2011).
